# Chronobiological Hypothesis about the Association Between Height Growth Seasonality and Geographical Differences in Body Height According to Effective Day Length

**DOI:** 10.5334/jcr.142

**Published:** 2016-11-11

**Authors:** Masana Yokoya, Yukito Higuchi

**Affiliations:** 1 Shimonoseki Junior College, 1–1 Sakurayama-cho, Shimonoseki, Yamaguchi 750-8508, Japan; 2 Kyushu Kyoritsu University, 1–8 Jiyugaoka, Yahatanishi-ku, Kitakyushu 807-8585, Japan

**Keywords:** Body height, Day length, Geographical difference, Height growth seasonality, DNA methylation, Epigenetics

## Abstract

Studies on growth hormone therapy in children have shown that height velocity is greater in summer than in winter and that this difference increases with latitude. It is hypothesized that summer daylight is a causative factor and that geographical distribution of body height will approximate the distribution of summer day length over time. This is an ecological analysis of prefecture-level data on the height of Japanese youth. Mesh climatic data of effective day length were collated. While height velocity was greatest during the summer, the height of Japanese youth was strongly and negatively correlated with the distribution of winter effective day length. Therefore, it is anticipated that summer height velocity is greater according to winter day length (dark period). This may be due to epigenetic modifications, involving reversible DNA methylation and thyroid hormone regulation found in the reproductive system of seasonal breeding vertebrates. If the function is applicable to humans, summer height growth may quantitatively increase with winter day length, and height growth seasonality can be explained by thyroid hormone activities that-induced by DNA methylation-change depending on the seasonal difference in day length. Moreover, geographical differences in body height may be caused by geographical differences in effective day length, which could influence melatonin secretion among subjects who spend a significant time indoors.

## Introduction

Recently, studies on children with growth hormone deficiency, receiving continuous exogenous recombinant human growth hormone (r-hGH) therapy, have shown that there is a seasonal variation in height growth at all latitudes, with summer height velocity being greater than winter height velocity [1,2,3]. This difference increased with distance from the equator and correlated with summer day length across different latitudes [1,2]. It has been reported that growth response was determined by an interaction between the summer day length and genes known to affect the growth response to r-hGH [1].

Many epidemiologic studies of nationwide data indicate that body height tends to increase at higher latitudes. These studies have shown the existence of a geographical (latitudinal) gradient in human body height [4,5,6,7,8,9]. A possible explanation for this gradient is the difference in photoperiodic environments [9]. Several potential mechanisms underlying the association between the photoperiodic environment and height in Japanese children have been reported. In a previous study, geographical differences in climatic variables, such as temperature, solar radiation, and effective day length (duration of photoperiod exceeding a predetermined light intensity) were analyzed, and it was shown that effective day length was the sole primary predictor of the geographical gradient in body height. The geographical variation in height has been attributed to differences in melatonin secretion due to variation in day length, inhibiting sexual and skeletal maturation [9].

Height velocity seasonality and geographical gradient of body height may both contribute to differences in height. However, this appears contradictory, as the phenomenon of height velocity seasonality suggests a positive relationship between summer day length and height growth velocity, while the phenomenon of geographical gradient of body height suggests a negative relationship between geographical difference in body height and annual day length. As regions with long summer days have short winter days, the two distributions are similar. However, the response of height growth to the photoperiod is opposite.

In previous ecological studies in Japan, effective annual day length was used to investigate the relationship between geographical differences in body height and the photoperiodic environment [9]. However, the association between geographical differences in body height and the distribution of seasonal day length were not considered. It would be prudent to examine the relationship between the geographical difference in body height and the geographical distribution of seasonal day length.

The aim of this study was to elucidate the association between geographical differences in body height and the photoperiodic environment in Japanese children and adolescents. Specifically, we focused on the seasonal effective day length. Effective day length is a photoperiod that takes into account the intensity of light; it becomes shorter with reduced solar radiation. As modern human beings spend the majority of their day indoors, relatively strong light and its duration will have an impact on biological processes.

According to the results, we considered the relationship between seasonal variation in height velocity and geographical differences in body height.

In this study, we investigated the geographical association between body height and seasonal effective day length in Japanese children and adolescents, using precise climatic data and the Geographic Information Science (GIS) technique.

## Materials and Methods

### Study area

This ecological study was conducted using prefecture-level data on Japanese children and adolescents. Supporting Information Figure S1 shows a map of the 47 prefectures in Japan [10], a long, thin archipelago with its longest axis oriented from north to south. Each prefecture was assigned a number (Supporting Information Tables S1–S4).

### Anatomical data

Prefecture-level anatomical data on Japanese children and adolescents were collected from the School Health Examination Surveys, conducted between 1989 and 2013 by the Ministry of Education, Culture, Sports, Science, and Technology. These surveys cover each of the 47 prefectures in Japan and contain data on mean height, weight, and other physical conditions, categorized according to sex and age (5 to 17 years) [11]. The survey on growth status uses a two-stage stratified random sampling method in which schools are classified by prefecture and type (stratification). A number of schools are then targeted (first-stage selection) and subjects (infants and students) are selected according to their age and sex, using a systematic sampling method (second-stage selection). The 2008 physical condition survey covered approximately 7800 schools and included approximately 700,000 students [11]. Sample size and original profiles are included in this database.

Prefecture-level mean weight and height data were collected. To eliminate annual fluctuations in values, 25-year (1989–2013) means of standardized values were calculated for each sex and age category using the following formula:

1 \[
   {Y_{ij}} = \frac{1}{{25}}\mathop \sum \limits_{k = 1}^{25} \frac{{{Z_{ijk}} - {u_{jk}}}}{{{\sigma _{jk}}}}
    \]

where *i* is the prefecture, *j* is the group (defined by age and sex), *k* is the year (1989–2013), *Zij* is the prefecture datum, *Yij* is the standardized datum over the 25-year period for each prefecture and sex standardized by mean, *µjk* is the national mean based on an analysis of individual data obtained from the survey reports, and *σjk* is the national-level standard deviation based on an analysis of individual data regarding age and sex across Japan in year *k* obtained from the survey reports [11].

The standardized height and weight for each prefecture are listed in Supporting Information Tables S1 and S2. The associations between standardized height and weight according to age are presented in Supporting Information Table S3.

**Figure 1:**
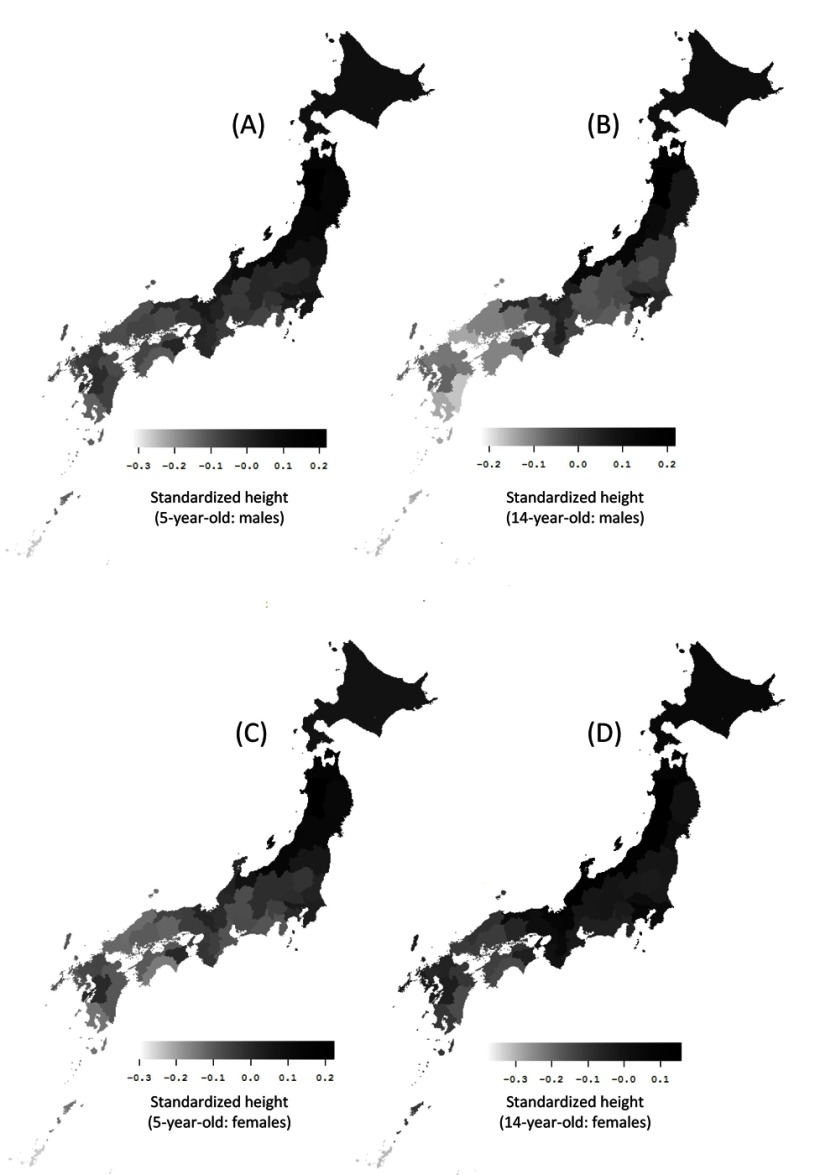
Distribution map of standardized height of Japanese youth. Distribution map of the 25-year (1989 to 2013) average of the standardized height in the following subjects, in each prefecture: **(A)** 5-year-old males, **(B)** 14-year-old males, **(C)** 5-year-old females, and **(D)** 14-year-old females. The body height of Japanese youth tends to be greater in northern prefectures.

Figure 1 maps the distribution of the mean standardized height in 5 and 14-year-old Japanese males and females over the 25-year study period and shows that height tends to be greater in Northern Japan and northern areas along the coast of the Sea of Japan.

### Climatic data

In the north, winter days are shorter than in the south and summer days are longer. On average, the possible sunshine duration is the same at all points of the globe. However, as modern human beings spend the majority of their time indoors, it is unlikely that differences in ambient possible sunshine duration have a direct impact on any biological process. Human photoperiodic sensitivity is often not sensitive enough [12,13] and the light intensity of indoor environments is generally less than 10 per cent of the outdoor light intensity [14,15] so relatively strong daylight will be considered.

In a previous study, effective day length was proposed as a climatic element indicating ambient daylight intensity and length [16]. Effective day length is a photoperiod that takes into account the intensity of light; it becomes shorter with decreases in solar radiation. The effective day length at more than 1000 lx increases in almost direct proportion to the intensity of solar radiation, and any effective day length is almost directly proportional to another at its intensity exceeding 1000 lx. The effective day length at 0 lx light intensity is equivalent to possible sunshine duration. Effective day length at any light intensity can be estimated by the empirical model, using solar radiation data [16]. First, monthly means of effective day length at 0 lx (possible sunshine duration), 1000 lx, and 5000 lx, were calculated. Moreover, the six-month average of the seasonal climatic value (*Savg*) was calculated using the following formula:

2 \[
   Savg = {\frac{\sum({M\times d})}{\sum ( d )}}
    \]

where *M* is the monthly average of effective day length, and *d* is the number of days in the month. The six-month average climatic value in the summer season was calculated using a value representing April to September, and the climatic value in the winter season was calculated using a value representing October to March.

The 25-year mean body size was calculated before considering the corresponding period of climatic data. However, as it is unclear whether the effects of climate are constant at all stages of growth, the use of mean climatic data from birth until the current age of each cohort may not have been useful in this analysis. Therefore, the analysis was performed using the climatic normal, defined as normal weather conditions calculated over a 30-year period. To improve analytical precision, mesh (region) climatic data for the year 2000 [17], which consist of climatic normal data, were obtained from the Japanese Meteorological Agency.

This dataset included monthly mean temperatures, solar radiation levels, hours of sunshine, precipitation levels, and snow cover for 1-km mesh areas. These data were based on observations performed between 1971 and 2000, with the climatic values in meshes with observing stations changed to observed values [17]. The monthly mean solar radiation data were used as input data to calculate the monthly value of effective day length.

**Figure 2:**
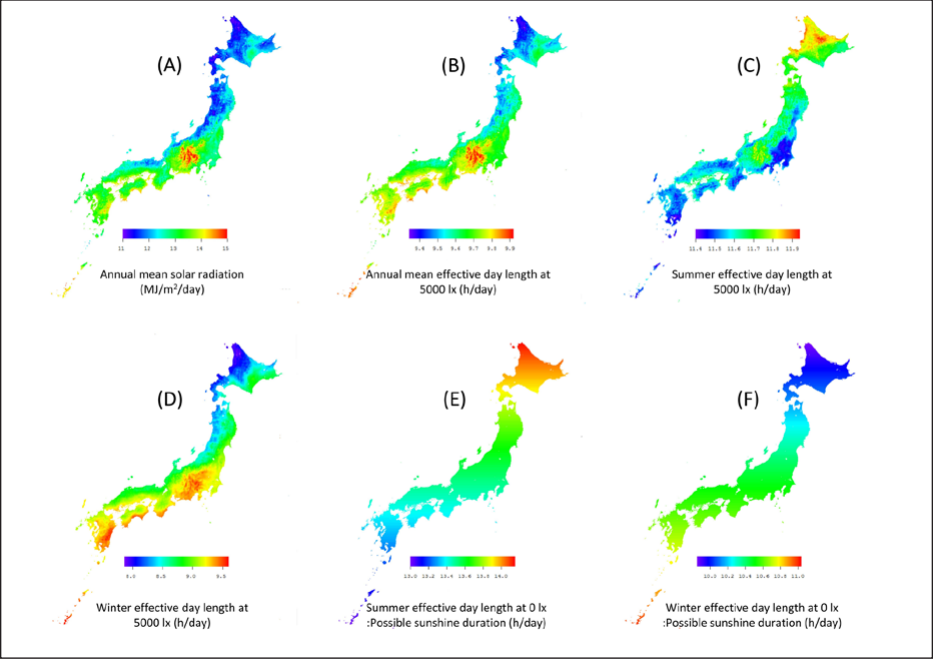
Distribution map of climatic variables in Japan. Mesh climatic data of 380,000 one–kilometer mesh areas are shown. The fill in the mesh areas indicates **(A)** annual mean solar radiation (MJ/m2/day), **(B)** annual mean effective day length at 5000 lx (h/day), **(C)** summer effective day length at 5000 lx (April to September: h/day), **(D)** winter effective day length at 5000 lx (October to March: h/day), **(E)** summer effective day length at 0 lx (possible sunshine duration) (h/day), and **(F)** winter effective day length at 0 lx (possible sunshine duration) (h/day). Annual mean solar radiation, annual mean effective day length at 5000 lx, and winter effective day length at 5000 lx, tend to be relatively low in the northern areas along the Sea of Japan.

The climate distribution in Japan is presented in Figure 2, which shows the Japanese mesh climatic data map of 380,000 1-km mesh areas, displaying (A) annual mean solar radiation (MJ/m^2^/day), (B) annual mean effective day length at 5000 lx (h/day), (C) summer effective day length at 5000 lx (April to September: h/day), (D) winter effective day length at 5000 lx (October to March: h/day), (E) summer effective day length at 0 lx (possible sunshine duration) (h/day) and (F) winter effective day length at 0 lx (possible sunshine duration) (h/day).

Because many meshes in Japan have no inhabitants, it was undesirable to obtain mean prefecture climatic values based on a simple calculation of the mean mesh value. Thus, there was a scale discrepancy between the high-resolution climatic data and prefecture-level anatomical data. To account for spatial differences, Mesh Population Data [18] from the year 2000, compiled from the results of the 2000 Population Census and produced under the same code and standards as the mesh climatic data, were used to calculate the population-weighted mean climatic value (*Cavg*) for each prefecture using the following formula:

3 \[
   Cavg = \frac{{\sum\nolimits_{n = 1}^m {(T \times P)} }}{{\sum\nolimits_{n = 1}^m {(P)} }}
    \]

where *m* is number of meshes in each prefecture, *T* is the climatic value in the mesh, and *P* is the population density in the mesh.

The population-weighted mean climatic values derived from the mesh data of each prefecture are listed in Supporting Information Table S4.

### Data analysis

Correlation analysis was performed using standardized height data and the population-weighted climatic value data (effective day length) for all 47 prefectures. The association between height and climatic data was further explored via multiple linear regression modeling. A linear regression model was employed to predict regional differences in height according to effective day length as follows:

4 \[
   {Y_i} = {k_0} + {k_1}{X}{1_i} + {k_2}{X}{2_i},
    \]

where *Y* is the linear predictor of the 25-year mean height for each prefecture in area *i, X1* is the 25-year mean weight for each prefecture in area *i, X2* is the population-weighted climatic value (effective day length) in area *i*, and *k1* and *k2* are the regression coefficients representing the effects of *X1* and *X2* on *Y*, respectively. In this case, weight was included to remove any bias caused by regional difference in eating habits, assuming that weight is an index of food intake (based on the assumption that the extent to which equilibrium weight is maintained is directly proportional to the level of food intake [19,20].

All statistical analyses were performed using R version 3.0.2 [21].

## Results

Table 1 shows the basic statistics regarding the height and weight of Japanese youth standardized over a 25-year period from 1989 to 2013. As can be observed, the maximum heights were observed in northern prefectures (Akita, Aomori), and the minimum heights were observed in southern prefectures (Okinawa, Miyazaki, Yamaguchi and Saga). Similarly, the maximum weights were observed in northern prefectures (Aomori and Akita), and the minimum weights were observed in southern prefectures (Okinawa and Yamaguchi).

**Table 1:** Basic statistics for standardized height and weight.

Standardized height	Males				Females			

Age	5	9	13	17	5	9	13	17
Maximum	0.214 Akita	0.224 Akita	0.237 Akita	0.166 Akita	0.221 Akita	0.241 Aomori	0.170 Akita	0.121 Akita
Minimum	–0.261 Okinawa	–0.248 Okinawa	–0.144 Miyazaki	–0.305 Okinawa	–0.202 Okinawa	–0.131 Yamaguchi	–0.287 Saga	–0.323 Okinawa
Mean	–0.009	–0.006	–0.005	–0.011	–0.003	0.004	–0.015	–0.013
Median	–0.014	–0.010	–0.020	–0.008	–0.010	–0.010	–0.016	–0.007
**Standardized weight**	**Males**				**Females**			

Age	5	9	13	17	5	9	13	17
Maximum	0.234 Aomori	0.295 Aomori	0.277 Aomori	0.220 Akita	0.242 Aomori	0.305 Aomori	0.253 Aomori	0.195 Akita
Minimum	–0.133 Okinawa	–0.116 Yamaguchi	–0.159 Yamaguchi	–0.139 Yamaguchi	–0.135 Okinawa	–0.114 Yamaguchi	–0.111 Yamaguchi	–0.254 Okinawa
Mean	–0.001	0.007	–0.001	0.002	0.003	0.012	0.010	0.014
Median	–0.031	–0.024	–0.017	–0.016	–0.031	–0.017	–0.008	0.002

Table 2 shows the results of the analysis of the 30-year average population-weighted climatic data (effective day length) in each prefecture. The maximum annual mean solar radiation and maximum annual mean effective day length at 0–5000 lx were observed in Koch. The minimum annual mean solar radiation and minimum annual mean effective day length at 1000–5000 lx were observed in Niigata. The maximum summer effective day length at 0–5000 lx was observed in Hokkaido, and the minimum summer effective day length at 0–5000 lx was observed in Okinawa and Tokyo. The maximum winter effective day length at 0–5000 lx was observed in Okinawa, and the minimum winter effective day length at 0–5000 lx was observed in Hokkaido and Akita.

**Table 2:** Basic statistics for climatic variables.

	SOLA	Ann 1000	Ann 5000	Summer 0	Summer 1000	Summer 5000	Winter 0	Winter 1000	Winter 5000

Maximum	14.00 Kochi	11.71 Kochi	10.50 Kochi	13.97 Hokkaido	13.52 Hokkaido	11.77 Hokkaido	10.98 Okinawa	10.68 Okinawa	9.50 Okinawa
Minimum	11.70 Niigata	11.61 Niigata	10.03 Akita	13.02 Okinawa	12.70 Okinawa	11.36 Tokyo	10.03 Hokkaido	9.72 Hokkaido	8.35 Akita
Mean	12.78	11.66	10.28	13.48	13.09	11.53	10.51	10.22	9.03
Median	12.80	11.66	10.30	13.47	13.09	11.52	10.53	10.23	9.12

SOLA: Annual mean solar radiation (MJ/m^2^/day). Ann 1000–5000: Annual mean effective day length at 1000 to 5000 lx (h/day). Summer 0–5000: Summer effective day length at 0 (possible sunshine duration) to 5000 lx (h/day). Winter 0–5000: Winter effective day length at 0 (possible sunshine duration) to 5000 lx (h/day).

Table 3 shows the correlation matrix of climatic variables (effective day length) as determined by Pearson’s correlation coefficient analysis of the set of population-weighted climatic values obtained in the 47 prefectures. Most of the climatic variables were significantly correlated with each other; however, the summer effective day length at 5000 lx only slightly correlated with annual mean solar radiation and annual mean effective day length at 1000–5000 lx.

**Table 3:** Pearson correlation matrix of climatic variables.

	SOLA	Ann 1000	Ann 5000	Summer 0	Summer 1000	Summer 5000	Winter 0	Winter 1000	Winter 5000

SOLA	1								
Ann 1000	0.95**	1							
Ann 5000	0.96**	0.96**	1						
Summer 0	–0.68**	–0.66**	–0.71**	1					
Summer 1000	–0.62**	–0.61**	–0.68**	0.99**	1				
Summer 5000	–0.10	–0.22	–0.33	0.60**	0.69**	1			
Winter 0	0.68**	0.66**	0.71**	–1.00**	–0.99**	–0.60**	1		
Winter 1000	0.78**	0.79**	0.83**	–0.98**	–0.97**	–0.61**	0.98**	1	
Winter 5000	0.84**	0.88**	0.95**	–0.80**	–0.80**	–0.61**	0.80**	0.90**	1

SOLA: Annual mean solar radiation. Ann 1000–5000: Annual mean effective day length at 1000 to 5000 lx. Summer 0–5000: Summer effective day length at 0 (possible sunshine duration) to 5000 lx. Winter 0–5000: Winter effective day length at 0 (possible sunshine duration) to 5000 lx. **p < 0.0001 *p < 0.005.

Table 4 shows Pearson’s correlation coefficients of the association between the height of 5 to 17-year-old subjects, standardized over the 25-year study period, and 30-year average values of several climatic variables. The correlation coefficient between height and weight was added as an index of food intake, on the assumption that the extent to which equilibrium weight is maintained is directly proportional to the level of food intake [19,20]. Height significantly correlated with all climatic variables, except summer effective day length at 5000 lx. The strongest correlation coefficient for annual mean effective day length at 5000 lx was found in 13-year-old male subjects (r = –0.89) and 12-year-old female subjects (r = –0.88). The correlation coefficient between height and weight was weaker for male subjects ≥14 years and female subjects ≥12 years.

**Table 4:** Pearson’s correlation coefficients of the relationship between height and climatic variables.

	SOLA	Ann 1000	Ann 5000	Summer 0	Summer 1000	Summer 5000	Winter 0	Winter 1000	Winter 5000	Weight

Males										
Age: 5	–0.82**	–0.78**	–0.82**	0.84**	0.80**	0.35	–0.84**	–0.87**	–0.80**	0.82**
6	–0.81**	–0.76**	–0.79**	0.80**	0.76**	0.29	–0.80**	–0.83**	–0.76**	0.82**
7	–0.82**	–0.78**	–0.82**	0.82**	0.78**	0.34	–0.82**	–0.85**	–0.80**	0.82**
8	–0.83**	–0.78**	–0.82**	0.83**	0.79**	0.33	–0.83**	–0.86**	–0.80**	0.81**
9	–0.84**	–0.80**	–0.84**	0.83**	0.80**	0.34	–0.83**	–0.87**	–0.82**	0.83**
10	–0.81**	–0.77**	–0.82**	0.84**	0.82**	0.37	–0.84**	–0.88**	–0.82**	0.86**
11	–0.77**	–0.74**	–0.80**	0.80**	0.78**	0.38	–0.80**	–0.84**	–0.80**	0.88**
12	–0.78**	–0.74**	–0.81**	0.80**	0.78**	0.38	–0.80**	–0.84**	–0.81**	0.87**
13	–0.83**	–0.79**	–0.86**	0.78**	0.76**	0.37	–0.78**	–0.84**	–0.85**	0.84**
14	–0.88**	–0.84**	–0.89**	0.77**	0.74**	0.32	–0.77**	–0.84**	–0.85**	0.78**
15	–0.88**	–0.85**	–0.87**	0.73**	0.69**	0.26	–0.73**	–0.80**	–0.82**	0.68**
16	–0.87**	–0.84**	–0.85**	0.72**	0.68**	0.24	–0.73**	–0.79**	–0.80**	0.66**
17	–0.86**	–0.83**	–0.83**	0.70**	0.65**	0.22	–0.70**	–0.77**	–0.78**	0.63**

Females										

Age: 5	–0.80**	–0.75**	–0.80**	0.82**	0.78**	0.35	–0.82**	–0.85**	–0.79**	0.84**
6	–0.78**	–0.74**	–0.78**	0.78**	0.74**	0.32	–0.78**	–0.81**	–0.76**	0.84**
7	–0.82**	–0.78**	–0.83**	0.81**	0.78**	0.34	–0.81**	–0.86**	–0.81**	0.83**
8	–0.81**	–0.79**	–0.84**	0.77**	0.75**	0.36	–0.78**	–0.83**	–0.83**	0.85**
9	–0.79**	–0.77**	–0.83**	0.74**	0.72**	0.38	–0.74**	–0.80**	–0.82**	0.86**
10	–0.76**	–0.74**	–0.81**	0.71**	0.70**	0.38	–0.71**	–0.78**	–0.81**	0.86**
11	–0.78**	–0.77**	–0.83**	0.73**	0.71**	0.38	–0.72**	–0.80**	–0.83**	0.78**
12	–0.86**	–0.83**	–0.88**	0.75**	0.72**	0.31	–0.75**	–0.82**	–0.84**	0.58**
13	–0.84**	–0.80**	–0.82**	0.70**	0.66**	0.23	–0.70**	–0.76**	–0.77**	0.49*
14	–0.87**	–0.83**	–0.84**	0.69**	0.65**	0.22	–0.69**	–0.77**	–0.78**	0.48*
15	–0.84**	–0.79**	–0.80**	0.65**	0.61**	0.17	–0.66**	–0.72**	–0.72**	0.49*
16	–0.81**	–0.77**	–0.76**	0.65**	0.60**	0.16	–0.65**	–0.71**	–0.70**	0.53*
17	–0.82**	–0.80**	–0.79**	0.65**	0.60**	0.19	–0.65**	–0.72**	–0.72**	0.56**

SOLA: Annual mean solar radiation. Ann 1000–5000: Annual mean effective day length at 1000 to 5000 lx. Summer 0–5000: Summer effective day length at 0 (possible sunshine duration) to 5000 lx (April to September). Winter 0–5000: Winter effective day length at 0 (possible sunshine duration) to 5000 lx (October to March). **p < 0.0001 *p < 0.005.

Table 5 shows Pearson’s correlation coefficients for the association between the weight of 5 to 17-year-old subjects, standardized over the 25-year study period, and 30-year average values of several climatic variables. Weight significantly correlated with all climatic variables, except summer effective day length at 5000 lx. However, among 11 to 14-year-old subjects, the correlation coefficient for the association between weight and the climatic variables was weak, in contrast to the correlation coefficient for the association between height and the climatic variables.

**Table 5:** Pearson’s correlation coefficients of the association between weight and climatic variables.

	SOLA	Ann 1000	Ann 5000	Summer 0	Summer 1000	Summer 5000	Winter 0	Winter 1000	Winter 5000	Weight

Males										
Age: 5	–0.56**	–0.51*	–0.55**	0.72**	0.70**	0.28	–0.72**	–0.70**	–0.55**	0.82**
6	–0.56**	–0.53*	–0.58**	0.72**	0.70**	0.34	–0.72**	–0.71**	–0.60**	0.82**
7	–0.58**	–0.55**	–0.61**	0.75**	0.74**	0.40	–0.75**	–0.75**	–0.65**	0.82**
8	–0.57**	–0.55**	–0.61**	0.74**	0.73**	0.39	–0.74**	–0.74**	–0.64**	0.81**
9	–0.58**	–0.55**	–0.61**	0.77**	0.75**	0.40	–0.76**	–0.76**	–0.65**	0.83**
10	–0.55**	–0.52*	–0.58**	0.76**	0.75**	0.38	–0.76**	–0.75**	–0.61**	0.86**
11	–0.51*	–0.48*	–0.54**	0.71**	0.71**	0.36	–0.71**	–0.70**	–0.58**	0.88**
12	–0.49*	–0.46*	–0.52*	0.71**	0.70**	0.38	–0.70**	–0.69**	–0.57**	0.87**
13	–0.52*	–0.48*	–0.55**	0.70**	0.70**	0.38	–0.70**	–0.69**	–0.59**	0.84**
14	–0.58**	–0.54**	–0.60**	0.73**	0.72**	0.37	–0.73**	–0.73**	–0.63**	0.78**
15	–0.60**	–0.58**	–0.61**	0.74**	0.72**	0.34	–0.74**	–0.74**	–0.62**	0.68**
16	–0.63**	–0.59**	–0.64**	0.76**	0.75**	0.35	–0.76**	–0.77**	–0.65**	0.66**
17	–0.65**	–0.62**	–0.66**	0.77**	0.75**	0.39	–0.77**	–0.78**	–0.69**	0.63**

Females										

Age: 5	–0.55**	–0.50*	–0.53*	0.70**	0.68**	0.28	–0.70**	–0.68**	–0.54**	0.84**
6	–0.54**	–0.52*	–0.57**	0.68**	0.67**	0.34	–0.68**	–0.68**	–0.59**	0.84**
7	–0.58**	–0.54**	–0.61**	0.71**	0.71**	0.37	–0.71**	–0.72**	–0.64**	0.83**
8	–0.56**	–0.54**	–0.61**	0.69**	0.68**	0.37	–0.69**	–0.70**	–0.63**	0.85**
9	–0.52*	–0.51*	–0.57**	0.66**	0.65**	0.38	–0.65**	–0.67**	–0.61**	0.86**
10	–0.49*	–0.48*	–0.53*	0.61**	0.60**	0.33	–0.61**	–0.62**	–0.56**	0.86**
11	–0.43*	–0.42*	–0.48*	0.59**	0.59**	0.32	–0.59**	–0.59**	–0.51*	0.78**
12	–0.37	–0.37	–0.43*	0.59**	0.59**	0.37	–0.59**	–0.57**	–0.48*	0.58**
13	–0.46*	–0.44*	–0.50*	0.67**	0.67**	0.40	–0.67**	–0.66**	–0.55**	0.49*
14	–0.50*	–0.48*	–0.53*	0.72**	0.72**	0.43*	–0.72**	–0.71**	–0.59**	0.48*
15	–0.53*	–0.53*	–0.55**	0.73**	0.71**	0.40	–0.73**	–0.72**	–0.59**	0.49*
16	–0.56**	–0.56**	–0.58**	0.74**	0.73**	0.42*	–0.74**	–0.74**	–0.63**	0.53*
17	–0.57**	–0.57**	–0.58**	0.75**	0.73**	0.42*	–0.75**	–0.74**	–0.63**	0.56**

SOLA: Annual mean solar radiation. Ann 1000–5000: Annual mean effective day length at 1000 to 5000 lx. Summer 0–5000: Summer effective day length at 0 (possible sunshine duration) to 5000 lx (April to September). Winter 0–5000: Winter effective day length at 0 (possible sunshine duration) to 5000 lx (October to March). **p < 0.0001 *p < 0.005.

Table 6 shows the results of multiple linear regression analysis performed to predict the height of 5-year-old Japanese children. The results indicate that all combinations of weight and climatic variables were significant predictors of height in 5-year-old subjects, except the summer effective day length at 5000 lx. A negative correlation was observed between height and annual mean effective day length and between height and winter effective day length. Conversely, a positive correlation was observed between height and summer effective day length. There was a small coefficient of determination (R^2^) in the combination of weight and summer effective day length.

**Table 6:** Regression coefficients (standard errors) of predictors of height in a 5-year-old child.

Male	Regression coefficient	Standard error	95% CI Lower	95% CI Upper	t	p	R^2^

Weight	0.52	0.07	0.382	0.656	7.65	<0.0001	0.859
SOLA	–0.53	0.07	–0.666	–0.392	–7.80	<0.0001	
Weight	0.57	0.07	0.431	0.709	8.26	<0.0001	0.842
Ann 1000	–0.48	0.07	–0.624	–0.346	–7.03	<0.0001	
Weight	0.53	0.07	0.397	0.663	8.02	<0.0001	0.862
Ann 5000	–0.53	0.07	–0.659	–0.393	–7.95	<0.0001	
Weight	0.44	0.10	0.247	0.640	4.54	<0.0001	0.796
Summer 0	0.52	0.10	0.321	0.714	5.30	<0.0001	
Weight	0.50	0.10	0.302	0.699	5.08	<0.0001	0.775
Summer 1000	0.46	0.10	0.257	0.654	4.62	<0.0001	
Weight	0.78	0.09	0.605	0.956	8.94	<0.0001	0.684
Summer 5000	0.13	0.09	–0.045	0.307	1.50	0.1399	
Weight	0.44	0.10	0.246	0.638	4.54	<0.0001	0.797
Winter 0	–0.52	0.10	–0.715	–0.323	–5.33	<0.0001	
Weight	0.41	0.08	0.240	0.577	4.88	<0.0001	0.840
Winter 1000	–0.58	0.08	–0.751	–0.414	–6.97	<0.0001	
Weight	0.54	0.07	0.396	0.680	7.64	<0.0001	0.845
Winter 5000	–0.50	0.07	–0.647	–0.363	–7.17	<0.0001	

**Female**							

Weight	0.58	0.06	0.454	0.712	9.12	<0.0001	0.871
SOLA	–0.48	0.06	–0.605	–0.347	–7.45	<0.0001	
Weight	0.62	0.06	0.494	0.752	9.70	<0.0001	0.861
Ann 1000	–0.44	0.06	–0.573	–0.314	–6.91	<0.0001	
Weight	0.58	0.06	0.463	0.707	9.65	<0.0001	0.882
Ann 5000	–0.49	0.06	–0.608	–0.364	–8.03	<0.0001	
Weight	0.53	0.09	0.351	0.719	5.85	<0.0001	0.811
Summer 0	0.44	0.09	0.256	0.624	4.82	<0.0001	
Weight	0.58	0.09	0.394	0.763	6.30	<0.0001	0.795
Summer 1000	0.39	0.09	0.207	0.576	4.27	<0.0001	
Weight	0.81	0.08	0.646	0.972	9.99	<0.0001	0.728
Summer 5000	0.13	0.08	–0.037	0.290	1.56	0.1251	
Weight	0.53	0.09	0.349	0.716	5.85	<0.0001	0.811
Winter 0	–0.44	0.09	–0.626	–0.259	–4.85	<0.0001	
Weight	0.50	0.08	0.337	0.657	6.27	<0.0001	0.849
Winter 1000	–0.51	0.08	–0.667	–0.347	–6.39	<0.0001	
Weight	0.59	0.06	0.461	0.720	9.17	<0.0001	0.868
Winter 5000	–0.47	0.06	–0.599	–0.339	–7.29	<0.0001	

SOLA: Annual mean solar radiation. Ann 1000–5000: Annual mean effective day length at 1000 to 5000 lx. Summer 0–5000: Summer effective day length at 0 (possible sunshine duration) to 5000 lx (April to September). Winter 0–5000: Winter effective day length at 0 (possible sunshine duration) to 5000 lx (October to March).

Table 7 shows the results of multiple linear regression analysis performed to predict the height of 9-year-old Japanese children. The results indicate that all combinations of weight and annual mean effective day length and all combinations of weight and winter effective day length were significant predictors of height in 9-year-old subjects. A negative correlation was observed between height and annual mean effective day length, and between height and winter effective day length; however, a positive correlation was observed between height and summer effective day length. The association in the combination of weight was not as significant as that for summer effective day length. The maximum determination coefficient (R^2^) of ≥ 0.90 is obtained in the combination of weight and annual mean effective day length at 5000 lx in females, indicating a strong agreement.

**Table 7:** Regression coefficients (standard errors) of predictors of height 9-year-old.

Male	Regression coefficient	Standard error	95% CI Lower	95% CI Upper	t	p	R^2^

Weight	0.52	0.06	0.397	0.643	8.51	<0.0001	0.889
SOLA	–0.54	0.06	–0.663	–0.417	–8.83	<0.0001	
Weight	0.56	0.07	0.430	0.699	8.45	<0.0001	0.861
Ann 1000	–0.49	0.07	–0.622	–0.353	–7.29	<0.0001	
Weight	0.51	0.07	0.374	0.651	7.47	<0.0001	0.868
Ann 5000	–0.52	0.07	–0.663	–0.386	–7.64	<0.0001	
Weight	0.47	0.11	0.259	0.688	4.45	0.0001	0.788
Summer 0	0.47	0.11	0.257	0.686	4.42	0.0001	
Weight	0.54	0.11	0.316	0.760	4.88	<0.0001	0.763
Summer 1000	0.39	0.11	0.171	0.615	3.57	0.0009	
Weight	0.83	0.09	0.650	1.012	9.27	<0.0001	0.696
Summer 5000	0.01	0.09	–0.172	0.189	0.10	0.9222	
Weight	0.47	0.11	0.259	0.686	4.45	0.0001	0.789
Winter 0	–0.47	0.11	–0.687	–0.259	–4.46	0.0001	
Weight	0.40	0.09	0.215	0.594	4.30	0.0001	0.832
Winter 1000	–0.57	0.09	–0.755	–0.376	–6.01	<0.0001	
Weight	0.52	0.08	0.359	0.685	6.46	<0.0001	0.830
Winter 5000	–0.48	0.08	–0.643	–0.317	–5.94	<0.0001	

**Female**							

Weight	0.62	0.06	0.505	0.732	11.01	<0.0001	0.896
SOLA	–0.46	0.06	–0.577	–0.351	–8.26	<0.0001	
Weight	0.63	0.06	0.515	0.751	10.82	<0.0001	0.886
Ann 1000	–0.45	0.06	–0.563	–0.328	–7.62	<0.0001	
Weight	0.57	0.06	0.461	0.683	10.37	<0.0001	0.908
Ann 5000	–0.50	0.06	–0.613	–0.391	–9.10	<0.0001	
Weight	0.66	0.09	0.478	0.840	7.33	<0.0001	0.793
Summer 0	0.31	0.09	0.126	0.488	3.41	0.0014	
Weight	0.67	0.09	0.491	0.858	7.41	<0.0001	0.785
Summer 1000	0.28	0.09	0.100	0.467	3.11	0.0032	
Weight	0.84	0.08	0.673	1.002	10.24	<0.0001	0.742
Summer 5000	0.06	0.08	–0.106	0.224	0.72	0.4762	
Weight	0.66	0.09	0.478	0.839	7.35	<0.0001	0.793
Winter 0	–0.31	0.09	–0.488	–0.127	–3.43	0.0013	
Weight	0.58	0.08	0.418	0.746	7.16	<0.0001	0.835
Winter 1000	–0.42	0.08	–0.580	–0.253	–5.12	<0.0001	
Weight	0.57	0.06	0.440	0.700	8.84	<0.0001	0.882
Winter 5000	–0.48	0.06	–0.606	–0.346	–7.38	<0.0001	

SOLA: Annual mean solar radiation. Ann 1000–5000: Annual mean effective day length at 1000 to 5000 lx. Summer 0–5000: Summer effective day length at 0 (possible sunshine duration) to 5000 lx (April to September). Winter 0–5000: Winter effective day length at 0 (possible sunshine duration) to 5000 lx (October to March).

Table 8 shows the results of multiple linear regression analysis performed to predict the height of 13-year-old Japanese adolescents. The results indicate that all combinations of weight and annual mean effective day length, weight and winter effective day length were significant predictors of height in 13-year-old males. The maximum determination coefficient (R^2^) of ≥ 0.90 was obtained in the combination of weight and annual mean effective day length at 5000 lx in males, indicating a strong agreement. However, combinations of weight and summer effective day length at 0–5000 lx were not significant predictors. In contrast to male subjects, multiple regression analysis was not as significant for females. Weight was not selected as a significant predictor of height.

**Table 8:** Regression coefficients (standard errors) of predictors of height 13-year-old.

Male	Regression coefficient	Standard error	95% CI Lower	95% CI Upper	t	p	R^2^

Weight	0.56	0.05	0.460	0.666	10.99	<0.0001	0.914
SOLA	–0.53	0.05	–0.637	–0.431	–10.43	<0.0001	
Weight	0.60	0.05	0.489	0.710	10.93	<0.0001	0.896
Ann 1000	–0.50	0.05	–0.609	–0.388	–9.09	<0.0001	
Weight	0.53	0.05	0.430	0.621	11.07	<0.0001	0.930
Ann 5000	–0.57	0.05	–0.664	–0.473	–11.98	<0.0001	
Weight	0.58	0.10	0.379	0.774	5.87	<0.0001	0.778
Summer 0	0.38	0.10	0.179	0.575	3.84	0.0004	
Weight	0.60	0.10	0.399	0.803	5.99	<0.0001	0.766
Summer 1000	0.34	0.10	0.141	0.545	3.42	0.0013	
Weight	0.82	0.09	0.642	0.993	9.38	<0.0001	0.708
Summer 5000	0.06	0.09	–0.116	0.235	0.68	0.5017	
Weight	0.58	0.10	0.379	0.774	5.88	<0.0001	0.778
Winter 0	–0.38	0.10	–0.575	–0.180	–3.85	0.0004	
Weight	0.50	0.08	0.327	0.666	5.89	<0.0001	0.834
Winter 1000	–0.50	0.08	–0.666	–0.327	–5.90	<0.0001	
Weight	0.52	0.06	0.400	0.644	8.61	<0.0001	0.893
Winter 5000	–0.54	0.06	–0.659	–0.415	–8.86	<0.0001	

**Female**							

Weight	0.13	0.09	–0.048	0.312	1.47	0.1477	0.715
SOLA	–0.78	0.09	–0.957	–0.597	–8.69	<0.0001	
Weight	0.17	0.10	–0.023	0.365	1.78	0.0826	0.663
Ann 1000	–0.72	0.10	–0.919	–0.531	–7.53	<0.0001	
Weight	0.10	0.10	–0.091	0.300	1.08	0.2877	0.681
Ann 5000	–0.77	0.10	–0.964	–0.572	–7.91	<0.0001	
Weight	0.04	0.14	–0.250	0.331	0.28	0.7825	0.484
Summer 0	0.67	0.14	0.378	0.959	4.63	<0.0001	
Weight	0.08	0.15	–0.220	0.386	0.55	0.5835	0.438
Summer 1000	0.60	0.15	0.300	0.907	4.01	0.0002	
Weight	0.47	0.14	0.186	0.758	3.32	0.0018	0.239
Summer 5000	0.04	0.14	–0.246	0.326	0.28	0.7788	
Weight	0.04	0.14	–0.250	0.330	0.28	0.7818	0.485
Winter 0	–0.67	0.14	–0.959	–0.379	–4.65	<0.0001	
Weight	–0.03	0.13	–0.285	0.229	–0.22	0.8259	0.585
Winter 1000	–0.78	0.13	–1.040	–0.527	–6.14	<0.0001	
Weight	0.09	0.11	–0.138	0.321	0.80	0.4265	0.593
Winter 5000	–0.72	0.11	–0.946	–0.486	–6.27	<0.0001	

SOLA: Annual mean solar radiation. Ann 1000–5000: Annual mean effective day length at 1000 to 5000 lx. Summer 0–5000: Summer effective day length at 0 (possible sunshine duration) to 5000 lx (April to September). Winter 0–5000: Winter effective day length at 0 (possible sunshine duration) to 5000 lx (October to March).

Table 9 shows the results of multiple linear regression analysis performed to predict the height of 17-year-old Japanese adolescents. The results indicate that none of the combinations of weight and effective day length were significant predictors of height in 17-year-olds. Weight was not significant as a predictor of height.

**Table 9:** Regression coefficients (standard errors) of predictors of height 17-year-old.

Male	Regression coefficient	Standard error	95% CI Lower	95% CI Upper	t	P	R^2^

Weight	0.13	0.10	–0.068	0.333	1.33	0.1890	0.741
SOLA	–0.77	0.10	–0.970	–0.569	–7.73	<0.0001	
Weight	0.18	0.10	–0.020	0.389	1.81	0.0763	0.715
Ann 1000	–0.72	0.10	–0.923	–0.514	–7.08	<0.0001	
Weight	0.14	0.11	–0.078	0.358	1.29	0.2029	0.704
Ann 5000	–0.74	0.11	–0.958	–0.521	–6.83	<0.0001	
Weight	0.23	0.17	–0.106	0.559	1.37	0.1764	0.508
Summer 0	0.52	0.17	0.190	0.855	3.17	0.0028	
Weight	0.32	0.17	–0.010	0.657	1.95	0.0571	0.469
Summer 1000	0.41	0.17	0.074	0.741	2.46	0.0178	
Weight	0.64	0.13	0.387	0.892	5.10	<0.0001	0.398
Summer 5000	–0.02	0.13	–0.276	0.230	–0.18	0.8553	
Weight	0.23	0.16	–0.106	0.558	1.37	0.1775	0.508
Winter 0	–0.52	0.16	–0.856	–0.192	–3.18	0.0027	
Weight	0.07	0.15	–0.233	0.381	0.49	0.6286	0.594
Winter 1000	–0.71	0.15	–1.018	–0.404	–4.66	<0.0001	
Weight	0.19	0.13	–0.068	0.441	1.47	0.1476	0.619
Winter 5000	–0.65	0.13	–0.902	–0.392	–5.11	<0.0001	

**Female**							

Weight	0.13	0.10	–0.069	0.339	1.33	0.1891	0.689
SOLA	–0.75	0.10	–0.950	–0.542	–7.37	<0.0001	
Weight	0.15	0.11	–0.064	0.365	1.41	0.1643	0.657
Ann 1000	–0.71	0.11	–0.929	–0.500	–6.71	<0.0001	
Weight	0.16	0.11	–0.067	0.378	1.40	0.1670	0.634
Ann 5000	–0.70	0.11	–0.919	–0.473	–6.29	<0.0001	
Weight	0.17	0.17	–0.170	0.515	1.02	0.3146	0.430
Summer 0	0.52	0.17	0.174	0.859	3.04	0.0040	
Weight	0.27	0.17	–0.072	0.612	1.59	0.1185	0.389
Summer 1000	0.40	0.17	0.057	0.741	2.35	0.0231	
Weight	0.59	0.14	0.312	0.859	4.31	0.0001	0.316
Summer 5000	–0.06	0.14	–0.335	0.213	–0.45	0.6545	
Weight	0.17	0.17	–0.172	0.513	1.00	0.3212	0.431
Winter 0	–0.52	0.17	–0.861	–0.176	–3.05	0.0038	
Weight	0.06	0.16	–0.254	0.374	0.39	0.7013	0.514
Winter 1000	–0.67	0.16	–0.985	–0.357	–4.30	0.0001	
Weight	0.17	0.13	–0.088	0.435	1.34	0.1881	0.542
Winter 5000	–0.61	0.13	–0.876	–0.353	–4.73	<0.0001	

SOLA: Annual mean solar radiation. Ann 1000–5000: Annual mean effective day length at 1000 to 5000 lx. Summer 0–5000: Summer effective day length at 0 (possible sunshine duration) to 5000 lx (April to September). Winter 0–5000: Winter effective day length at 0 (possible sunshine duration) to 5000 lx (October to March).

## Discussion

This study has focused on the geographical differences in body height associated with photoperiodic environment among Japanese children and adolescents. The results of correlation analyses indicated that height was negatively correlated with annual and winter effective day lengths. However, height correlated positively with summer effective day length. Height correlated more strongly with annual and winter effective day length than with summer effective day length. These findings, confirmed by multiple regression analysis, indicate that the combination of weight and annual or winter effective day length were more important predictors of height in children and early adolescents. Conversely, the significance level of summer effective day length was weaker than annual or winter effective day length.

These results indicate that the geographical distribution of body height among Japanese children and adolescents matches the geographical distribution of annual or winter days more closely than summer day length, even taking into account the difference in the light intensity of day length or differences in body weight. The distribution of body height among Japanese youth tends to be greater in the northern areas along the Sea of Japan and is tilted to the latitude line. This distribution is similar to the annual mean effective day length or winter effective day length. As cold currents from Siberia bring a heavy snow to Japan’s seaside in winter, the annual effective day length and winter effective day length tend to be low in the northern areas along the Sea of Japan. In brief, these distributions are characterized by winter weather.

In studies of children with growth hormone deficiency receiving continuous exogenous r-hGH therapy, a seasonal variation in growth at all latitudes has been shown, with summer height velocity exceeding winter height velocity; this difference also increased with distance from the equator and correlated with summer day length across different latitudes [1,2]. The interaction between the summer day length and genes known to affect the growth response to r-hGH has been reported [1].

It is generally accepted that height velocity in childhood is greatest during the summer, and slightest during the winter [2,3,22,23]. There are also reports that height velocity in Japanese children was greater in summer than in winter [24,25]. Therefore, this height growth seasonality appears to be a universal phenomenon.

Height velocity is greatest during the summer and differences in height velocity increase with distance from the equator. It follows that geographical distribution of body height will become closer to the distribution of summer day length over time. However, in this study, the geographical distribution of body height in children and adolescents more closely matched the distribution of annual or winter effective day length rather than summer effective day length. The two phenomena appear to be contradictory.

Assuming that both of these phenomena (height velocity is greatest in summer and the geographical distribution of body height follows the distribution of annual or winter day length) are supported by evidence, summer height velocity should be greater according to winter day length (dark period). It is therefore necessary to consider the function that can sustain the quantitative photoperiodic memory during a 6-month period. These phenomena may be explained by epigenetic modifications, by means of reversible DNA methylation and thyroid hormone regulation, found in the reproductive system of seasonal breeding vertebrates.

In mammals, melatonin is the hormonal relay for the photoperiodic message, governing thyroid-stimulating hormone (TSH) production. TSH acts on neighboring hypothalamic cells known as tanycytes, which in turn control hypothalamic function through effects on thyroid hormone signaling, mediated by changes in expression of the deiodinase type II and III (DIO2 and DIO3, respectively) [26]. Although thyroid secretion of the prohormone T4 does not change seasonally [27], hypothalamic expression of deiodinase enzymes catabolizing T4 into the receptor-active triiodothyronine T3 (DIO2) or the receptor-inactive enantiomer (DIO3) are regulated by changes in photoperiod [28,29,30,31,32], providing a seasonal gating mechanism for thyroid hormone receptor signaling. Winter photoperiods elevate *dio3* expression, quench T3 signaling, and inhibit gonadotropin secretion, whereas spring/summer photoperiods elevate *dio2* expression, enhance T3 signaling, and stimulate gonadotropin release [28,29,30,31,32,33].

Recently, it was reported that reversible DNA methylation and thyroid hormone regulation controlled seasonal reproductive processes in Siberian hamsters. In long-day breeding hamsters, exposure to winter photoperiods, or winter-like patterns of melatonin, altered DNA methyltransferase expression; this led to decreased DNA methylation in the proximal promoter region of DIO3 (*dio3*) and increased hypothalamic *dio3* expression. Pharmacological blockade of photoperiod driven demethylation attenuated reproductive responses to winter photoperiods. Winter demethylation was reversed in anticipation of spring in that spontaneous reproductive development was accompanied by re-methylation of the *dio3* promoter and decreases in *dio3* mRNA [34]. This supports the idea that DNA methylation states can provide the basis for the quantitative accumulation of photoperiodic memory [35].

There are various pathways or functions through which photoperiod or melatonin secretion affect reproductive or somatic regulation in mammals. For example, melatonin is related to the glucocorticoid receptor pathway and there is a well-established link between the circadian cortisol cycle and growth rate [36,37]. More recently, melatonin was implicated in gonadotropin regulation and glucose metabolism/insulin sensitivity [38,39,40]. However, these mechanisms are not supported as methods of expressing the past photoperiodic memory.

The function of reversible DNA methylation and thyroid hormone regulation are useful to quantitatively explain the mechanisms preserving past photoperiodic memory. Assuming that reversible DNA methylation and thyroid hormone regulation are applicable to humans, both phenomena (height velocity being the greatest in the summer season and geographical distribution of adolescent body height being negatively correlated with distribution of annual effective day length) can be explained with no contradiction.

If the function of reversible DNA methylation and thyroid hormone regulation is sufficiently robust to preserve past photoperiodic memory, T3 signaling in summer will be enhanced in accordance with the previous winter day length (dark period). Seasonal height velocity gradually increased with distance from the equator in subjects receiving of r-hGH therapy [1,2], therefore, it is conceivable that the function of reversible DNA methylation and thyroid hormone regulation is strikingly quantitative for preserving past photoperiodic memory in humans as well.

Similarly, if we apply the function of reversible DNA methylation and thyroid hormone regulation to humans, height velocity seasonality in r-hGH therapy and its geographical difference can be explained by thyroid hormone activity. Thyroid hormones are necessary for normal growth in children and young animals, as evidenced by growth retardation observed in thyroid deficiency. The growth-promoting effect of thyroid hormones is intimately intertwined with growth hormone, a clear indication that complex physiologic processes, like growth, depend upon multiple endocrine controls [41]. Thyroid hormone is often used in combination in a growth hormone therapy, and it is also used for the treatment of hypothyroidism. Generally, the prohormone T4 formulation is used for therapy because it is thought that the T4 formulation is converted to the T3 formulation in the body [42]. The seasonal change in the metabolic rate between the prohormone T4 and triiodothyronine (T3) is widely recognized, including in humans [43,44].

According to the function of reversible DNA methylation and thyroid hormone regulation, T3 signaling in summer will increase in the region where there is a short winter photoperiod (long dark period). However, annual total possible sunshine duration is constant across geographical areas. Even accounting for the seasonal distribution of possible sunshine duration, the distribution is latitudinal. However, in order to understand the regional differences in height and geographical differences in photoperiod, it is necessary to adapt the concept of effective day length to explain the geographical difference in the photoperiodic environment. As modern humans spend much time indoors, the dark period is prolonged more than that in the outdoor natural environment [14,15]. This effect becomes more pronounced in high latitude regions experiencing a relatively weak intensity of daylight.

Previous research has shown that the light intensity of indoor environments is generally less than 10 per cent of outdoor light intensity [14,15] and that the annual total possible sunshine duration is constant across geographical areas. When these facts are considered together, geographical differences in effective day length at low light intensities (<1000 lx) are too small to produce a geographical gradient in body height (the geographical difference between the two extreme values in annual mean effective day length at 1000 lx is only 0.10 h/day (Table 2)). Exposure to light intensity greater than 5000 lx appears to be capable of explaining the geographical difference in body height (the geographical difference between the two extreme values in the annual mean effective day length at 5000 lx is 0.47 h/day (Table 2)). In this case, we cannot say a fixed threshold of light intensity would have a sufficient impact to induce a geographical difference in body height, because the threshold changes with a room structure or an individual’s light sensitivity and several other factors. However, since the distribution of solar radiation and any threshold of effective day length are almost proportional to each other, as a result, any threshold of effective day length becomes longer in regions receiving greater solar radiation in term of probability. Therefore, the individual level difference is offset in the population-based study.

In seasonally breeding vertebrates, the function of reversible DNA methylation and thyroid hormone regulation is indispensable for reproduction. In this study, the distribution of height was closer to the distribution of effective day length in adolescents (14-year-old males, 12-year-old females); this may imply that there is a relationship with sexual maturation. Photoperiodic environment might somehow affect human sexual maturation. However, a previous study analyzing population-based cross-sectional growth curves in Japanese prefectures did not show the geographical difference in age at Peak Height Velocity [10]. Moreover, the biological merit of this function or the advantage of inducing a geographical difference in body size is unknown. It may be only a vestige of seasonal breeding rather than an adaptation to a natural environment. The geographical difference in body height should not occur under the natural outdoor photoperiodic environment; however, it may be associated with the unexpected change caused by the human habit of living indoors.

Ecological analysis of nationwide data indicates that the body height of Japanese youth tends to be greater in Northern Japan over the past 100 years or more, suggesting that there exists a geographical gradient in youth height over this period [7,8,9]. Although the physique of Japanese youth was very poor at the end of World War II, rapid economic growth and strenuous efforts to improve nutritional status have led to great improvements in the physical development of Japanese youth since 1945. The Ministry of Education, Culture, Sports, Science, and Technology reported that after peaking between 1997 and 2001, the physical development of the average youth has remained at a level much higher than that observed in the history of Japan [45]. Nevertheless, a geographical gradient in body height is still observed. Examination of the nutritional improvement and economic growth occurring during this period indicates that it is probably the result of environmental rather than nutritional or socioeconomic factors. This is the primary reason for suspecting climatic impact for regional differences in body height. However, we cannot deny alternative explanations for these observations. Caloric intake, exercise habits, and environmental, socioeconomic, and genetic factors, are some of the factors that might play a role in geographical differences in body height.

Except for Japan, little research has been conducted on the geographical correlation between day length and human body height that may reflect the belief that nutritional and/or genetic factors are strongly related to body height. Compared to other countries, the Japanese population exhibits few geographical differences in nutritional intake [46,47,48] and is relatively ethnically homogeneous, appearing to have less genetic variation than populations of other countries [49]. This nutritional and genetic uniformity may be responsible for the ability to identify the effects of day length on body height and the existence of a geographical gradient in body height in Japan.

All ecological studies are prone to ecological fallacy, and this study offers only a partial explanation for geographical differences in body height in Japanese adolescents and children. Therefore, additional studies using individual-level data to evaluate the impact of photoperiodic factors on anatomical factors should be performed. In addition, the physiological mechanism has not been definitively identified. Specifically, the epigenetic function of DNA methylation in annual breeders, including humans, is not fully recognized [34,35]. Further research is necessary to definitively establish the association between climatic factors and health/anatomical factors.

## Conclusions

Despite height growth velocity being greatest during the summer, the distribution of the height of Japanese children and adolescents was strongly negatively correlated with the distribution of annual or winter effective day length.

Studies have demonstrated that summer height velocity was quantitatively greater according to winter day length (dark period). One hypothesis explaining this phenomenon is the epigenetic state, capable of quantitatively preserving past photoperiodic memory. This vernal phenomenon has been observed in the reproductive functions of several vertebrates. In mammals, reproductive development of seasonally breeding vertebrates is controlled by reversible DNA methylation and thyroid hormone regulation.

If reversible DNA methylation and thyroid hormone regulation are applicable to humans, summer height growth may be greater quantitatively in accordance with winter day length (dark period). Moreover, height growth seasonality can be explained by the thyroid hormone activities induced by the reversible DNA methylation status derived by the change in day length or the duration of melatonin secretion. Further, geographical differences in body height may be caused by geographical differences in effective day length and relatively strong light intensity could influence the secretion of melatonin of humans who spend the majority of their time indoors.

## Additional Files

The additional files for this article can be found as follows:

- **Additional File 1: Figure S1. **jcr-14-142-s1.eps
- **Additional File 2: Table S2. **jcr-14-142-s2.xls
- **Additional File 3: Table S3. **jcr-14-142-s3.xls
- **Additional File 4: Table S4. **jcr-14-142-s4.xls
- **Additional File 5: Table S5. **jcr-14-142-s5.xls
